# Illness Mapping: a time and cost effective method to estimate healthcare data needed to establish community-based health insurance

**DOI:** 10.1186/1471-2288-12-153

**Published:** 2012-10-09

**Authors:** Erika Binnendijk, Meenakshi Gautham, Ruth Koren, David M Dror

**Affiliations:** 1 Institute of Health Policy and Management, Erasmus University Rotterdam, P.O. Box 1738, Rotterdam, 3000 DR, the Netherlands; 2 Felsenstein Medical Research Centre, Tel Aviv University Sackler Faculty of Medicine, Ramat Aviv, Tel Aviv, Israel; 3 Micro Insurance Academy, 52B Okhla Industrial Estate Phase III, New Delhi, 110020, India

**Keywords:** India, Community based health insurance (CBHI), Micro health insurance, Illness prevalence, Incidence of hospitalization, Illness Mapping

## Abstract

**Background:**

Most healthcare spending in developing countries is private out-of-pocket. One explanation for low penetration of health insurance is that poorer individuals doubt their ability to enforce insurance contracts. Community-based health insurance schemes (CBHI) are a solution, but launching CBHI requires obtaining accurate local data on morbidity, healthcare utilization and other details to inform package design and pricing. We developed the “Illness Mapping” method (IM) for data collection (faster and cheaper than household surveys).

**Methods:**

IM is a modification of two non-interactive consensus group methods (Delphi and Nominal Group Technique) to operate as interactive methods. We elicited estimates from “Experts” in the target community on morbidity and healthcare utilization. Interaction between facilitator and experts became essential to bridge literacy constraints and to reach consensus.

The study was conducted in Gaya District, Bihar (India) during April-June 2010. The intervention included the IM and a household survey (HHS). IM included 18 women’s and 17 men’s groups. The HHS was conducted in 50 villages with1,000 randomly selected households (6,656 individuals).

**Results:**

We found good agreement between the two methods on overall prevalence of illness (IM: 25.9% ±3.6; HHS: 31.4%) and on prevalence of acute (IM: 76.9%; HHS: 69.2%) and chronic illnesses (IM: 20.1%; HHS: 16.6%). We also found good agreement on incidence of deliveries (IM: 3.9% ±0.4; HHS: 3.9%), and on hospital deliveries (IM: 61.0%. ± 5.4; HHS: 51.4%). For hospitalizations, we obtained a lower estimate from the IM (1.1%) than from the HHS (2.6%). The IM required less time and less person-power than a household survey, which translate into reduced costs.

**Conclusions:**

We have shown that our Illness Mapping method can be carried out at lower financial and human cost for sourcing essential local data, at acceptably accurate levels. In view of the good fit of results obtained, we assume that the method could work elsewhere as well.

## Background

A large part of health care spending in developing countries is private and out of pocket (OOP). India is typical: 70% of spending is private, of which 86% is OOP [1,2]. Moreover, private insurance rates remain below 5% [3]. The dearth of insurance is surprising, given the high frequency and cost of borrowing from moneylenders even for outpatient care and maternity [4] in addition to inpatient care [4,5], and the inability of rural poor to pay for non-communicable diseases [6] even as the prevalence of NCDs increases in low-income countries [7,8]. One possible explanation for low insurance penetration is that poorer individuals in the informal sector doubt their ability to enforce contracts with insurance companies. A solution to the problem is community-based health insurance schemes (CBHI) [9-12]. These schemes are owned and run locally, at village level [12,13]. One of the hurdles to launching CBHI schemes is obtaining relevant information on local morbidity, healthcare utilization and other information that would inform the design and pricing of a relevant and affordable insurance package. A number of experiments with micro health insurance have relied on household surveys to obtain reliable local actuarial estimates and other information required for package design and pricing [14-16]. Obtaining accurate local data is essential both because the income of CBHI is often limited and because of significant differences across locations in the number and type of illness episodes [17-19]. However, household surveys are both expensive and time consuming. Thus a faster and cheaper method would be instrumental in promoting the expansion of micro health insurance.

Our study is located in Gaya district, Bihar state, India. The main source of data on incidence/prevalence of illnesses and hospitalisations is the Indian National Sample Survey (NSS) [20]. The NSS however provides information only at state level and not at district or block level, which are the more relevant units for CBHI. In addition, the most recent edition of NSS with information on morbidity and healthcare utilization dates to 2004 [20] with an earlier survey in 1995/96 [21]. And, health information sourced from local medical record-keeping does not provide sufficiently accurate location-specific data.

This paper contains a description of a cheaper and faster method to derive quantitative estimates of healthcare events through qualitative approaches [22]. The experiment we conducted is inspired by previous methodologies aiming to achieve similar objectives. For instance, Auray and Fonteneau [23] suggested possible group methods using consensus-building techniques, notably the Delphi and the Nominal Group Technique (NGT), to derive estimates from expert opinions on prevalence of hospitalizations, incidence of illness etc.

In the Delphi method, individual experts that are not in contact with each other first provide their quantitative estimate to a query; then, each expert is informed about other experts’ replies, and invited to adjust the value (but each expert does so alone, without interacting with the others); this process can be repeated several iterations until consensus is reached [22,24]. In the NGT, experts that are assembled in the same place at the same time individually write down their views on the topic in question and present one idea to the facilitator which is recorded. There is a group discussion to clarify and evaluate each idea and following this discussion each participant privately ranks each idea. This ranking is tabulated and presented. The group then discusses the overall ranking to reach consensus [22,25,26]. It is noted that while there is some interaction between NGT group members to discuss or clarify ideas, other major group processes, such as idea generation and final rankings, are conducted silently and individually [26]. So, while both the Delphi and NGT are methods to reach consensus, both unfold among *non-interacting groups* (participants do not interact and discuss with each other during the group process) [26,27]. In interacting groups on the other hand, participants are allowed to interact and discuss with each other at each step of the process (generation of information, ideas, views, evaluation and final consensus) [27]. Interacting groups are usually unstructured (participants have complete freedom to think, review and synthesize together); examples are Brainstorming discussions and Focus Group Discussions [28]. Non-interacting groups however, are usually structured (participants receive systematic procedural guidance) [24,26].

Research in the 1960s and 70s compared non-interacting groups with interacting groups [26,27,29,30]. Delphi and NGT have been found superior to interacting groups for finding solutions to problems [26], but when group interactions were structured to enhance exchanges among the participants during thinking, visualizing and estimating, results were better than with unstructured interactions [31,32]. Moreover, Van de Ven and Delbecq [27] found that the most optimal group processes occurred when a structured procedure entailed interactive discussions after the initial exposé of ideas/views. Bouchard [30] found that group-results were enhanced when the groups consisted of carefully selected individuals who had some prior knowledge of each other and some practice of working or being together (where differences that might inhibit group effectiveness were minimized).

Our study entailed a variation of an interactive group technique, inspired by the non-interactive group techniques. We elicited expert opinion in which our experts were members of the target community that knew each other, whose opinions were obtained in a structured, interactive group situation. The purpose of the inquiry has been to derive estimates of healthcare data needed to establish micro health insurance. We call this method “Illness Mapping”. With the view to verifying robustness of results of the Illness Mapping method, we compared them to household survey data from the same locations and period. Our working assumption was that if the Illness Mapping delivered useful comparator data in this case, this method could be used elsewhere as an alternative to household surveys for faster and cheaper resourcing of the context-relevant essential data.

## Methods

### Setting and sampling

The study was conducted in Gaya District of Bihar state, India. Gaya district is subdivided into 24 blocks. We selected 7 contiguous blocks purposively because this is where a local partner Non-governmental Organization (NGO) intended to implement a micro health insurance scheme. The intervention included two exercises: the Illness Mapping and a household survey. Both activities were conducted during April-June 2010.

For the Illness Mapping, we divided the 7 blocks into 3 clusters (northern, middle and southern) and selected 6 villages in each cluster based on distance from the nearest government primary health centre (0–5 kms; 5.1-8 kms; and more than 8 kms). Our total sample included 18 villages, (7 villages in the 0–5 kms category; 6 villages in the 5.1-8 kms category; and 5 villages in the >8 kms category). In consultation with the field partner, we selected a male group and a female group in each village, each with about 10 participants. The groups were gender homogenous to enable participants to speak freely on the given subject. There were 18 women’s groups (263 participants) and 17 men’s groups (147 participants).

The household survey was conducted in 50 villages across Gaya district, selected randomly (using census list of villages) from all 24 blocks in the district, proportional to the number of villages in each block. Within each village, we interviewed 20 households, selected randomly by applying the “four winds technique”, or “line sampling” (selecting households according to a predetermined staggering e.g. every second/third household starting from the centre of the village and progressing in the four cardinal directions) [33]. In total, 1,000 households were interviewed, representing 6,656 individuals.

Verbal informed consent was obtained from respondents of the household survey at the beginning of the interviews, and from participants of the Illness Mapping before the discussions began. 100% of the interviewed sample was rural.

### Illness mapping

The Illness Mapping technique is an adaptation of two non-interactive consensus group methods (Delphi process and Nominal Group Technique – NGT) operated in an interactive manner. The adaptation was necessary because it was impossible to apply the Delphi and NGT as is (i.e. sending our experts a questionnaire and/or requesting each to write ideas individually) due to the limited literacy of the population. Rather, interaction between the facilitator and the group members became essential, especially as the option of reaching decisions by vote was discarded, in light of the finding in one of our previous studies in India that rural participants preferred to reach a consensus [34].

Like the Delphi and NGT techniques, Illness Mapping relies on the knowledge of experts. Prior to the selection of the experts, our research team met with key informants in the village [health/development workers such as the Accredited Social Health Activist (ASHA), Aanganwadi Worker (AWW) or Auxiliary Nurse Midwife (ANM), representatives of Self Help Groups, etc.] to get an overview of the village, its size, social segmentation, and a general impression of its socio-economic status. Using this knowledge, we selected our experts by applying the following criteria:

1. They should be living in different parts of the village.

2. They should be sociable, outgoing and interacting frequently with their neighbours, so that they would be knowledgeable about people and events in the village. Not surprisingly, participants with higher interpersonal skills have been found to perform better in group discussions [30].

3. Group members should reflect similar social or income groups.

In the Illness Mapping facilitators (of the same gender as the participants) guided group meetings to enhance recall of the parameters needed for the calculation of the prevalence of illnesses and utilization of health services. Such facilitated recall procedure does not occur either in the Delphi or the NGT, but publications suggested that compared to unstructured interventions, participants recall the relevant parameters better when procedures are structured during the thinking, visualizing and estimating stage of the interaction with the facilitator [31,32]. Considering that people with motivation or training have been reported to perform better in group interactions [30], we motivated our participants by explaining that they were selected for this discussion from the entire village, and that the information they provided would help develop the right kind of health insurance benefits for them and the entire village.

With each group, we first obtained a rough estimate of the number of households in different parts of the village, the rough household size (i.e. number of family members that ate from the same pot), and the total population of the village. Then we asked the number of persons who had been sick over the last one month, and the nature of their illness. We then asked every participant to name, one after the other, all the illnesses they could remember. To facilitate recall, the facilitator prompted periodically by asking about specific illnesses by name, both common and not so common ones. We also enquired about incidence of hospitalizations and deliveries (during the last 12 months) including information whether the delivery occurred at home or in an institute.

Consensus was reached through a structured group discussion of the final tallies, similar to the final round of the NGT. We presented to each group the final tallies of the main illness categories and frequencies of illnesses, hospitalizations and deliveries, and asked for feedback on the illness tallies (presented both as a number and as a percentage of the total village population). Usually participants chose to increase the final cumulative percentage. In the few instances where the group was not able to arrive at a single estimate, we noted the different estimates (usually 2–3 different estimates) and averaged them.

Similar to the Delphi method, our facilitator combined all responses and fed those back to the experts, who then ranked all opinions/solutions to obtain a new “agreed value”, which was again combined and distributed. Like in the Delphi, the experts can re-evaluate their ranking and possibly change their original opinions/solutions [22].

As in NGT, our Illness Mapping process occurs in a meeting. And, like NGT interaction is limited in the first part of the process when each expert gives their response to the facilitator (in NGT this is done in writing). A group discussion follows, to clarify and evaluate responses, and reach consensus (in NGT, unlike our Illness Mapping, before discussion to reach consensus each expert ranks responses separately, and the ranking is tabulated and presented) [22,25].

Data obtained in group discussions were recorded on pre-designed data sheets; a second person, other than the facilitator recorded the responses. Names and frequencies of illnesses^i^ were recorded; we classified the illnesses reported as acute, chronic, accidents, and undefined. 18 groups from 14 villages provided 8 or more names of illnesses; only these groups were retained for the analysis of illness types. Hospitalizations and deliveries were counted and presented separately.

### Household survey

The household survey questionnaire included questions on general demographics (age, gender, education, economic activity), socio-economic status (queried through questions on many items of household expenditures) and health status of household members. Following the method of the Indian National Sample Survey Organization [35], we consider the monthly per capita consumer expenditure excluding healthcare costs as a proxy for income. Respondents were asked about illness episodes in the household during the month preceding the survey. Using the replies regarding the illness (related to symptoms, length of illness, recurrence, medication etc.), we classified illnesses into four categories: acute, chronic, accidents and undefined. Respondents were asked about hospital admissions in the year preceding the survey and deliveries in the two years preceding the survey including where the delivery took place (home or hospital). The household survey questionnaire was translated into Hindi (the local language), back translated for validation, and pre-tested among 80 households in the area. Surveyors who spoke the local language fluently conducted the survey.

### Data presentation and statistical analysis

We used Stata (version 11) for a descriptive analysis of the household survey. We used MS Excel (version 2003) for the Illness Mapping data tabulation and analysis.

The incidence of illness and health care utilization derived from the household survey are represented in percentages by dividing the number of cases by the overall number of members of the sampled households. The estimates derived from the Illness Mapping are presented as the mean and standard error of the mean (SEM) of all the group estimates arrived through consensus (male/female groups separately and all groups). We compared information obtained from male vs. female groups to ascertain that familiarity with local illnesses was comparable, and significance of this difference was assessed by Student’s *t*-test. When comparing the results from the Illness Mapping with the results from the household survey we considered as “good fit” results of the Illness Mapping that were less than two SEM of the household survey data and as “very good fit” the results that were less than one SEM.

## Findings

### Socioeconomic and demographic profile of the sampled population

The information on socioeconomic and demographic status of the sampled population in Gaya (one of the districts of Bihar state) is summarized in Table 1. As can be seen, the population is resource-poor (income is about PPP$ 1.53 per person per day), poorly educated (44% with no schooling whatsoever), and the main source of earning is daily wage labour (60%) and self-employed in agriculture (24%). As a comparison, monthly per capita consumer expenditure (not including medical expenditures) was INR 753 in rural Bihar according to NSS (=PPP$ 1.39 per person per day) [36].

**Table 1:** Socioeconomic and demographic information obtained

	
	Mean (± SE^a^)
Income-proxy per person per month^b^ (INR)	832.62 (± 7.05)
Household size	7.97 (± 0.04)
	Share of population
Education of population (15 years and older)	
No schooling	43.67%
Class 1-5	12.08%
Class 6-10	34.55%
Class 11 and higher	9.69%
Economic activity of income earners (15 years and older)	
Daily wage labourer	60.43%
Self-employed in agriculture	24.30%
Self-employed in business/trade	7.89%
Regular salaried employee	7.38%

### Prevalence of illnesses

Local prevalence of illnesses is one of the main parameters for designing and pricing health insurance. We compared the estimate of prevalence of illnesses (the percentage of persons ill in the last month) from the Illness Mapping methodology with the conventional household survey (Table 2). The comparison of the mean value of prevalence of illness obtained through the Illness Mapping and that obtained through the household survey were less than two SEM, and provided “good fit”. Furthermore, the results obtained from groups composed of males and females were not significantly different from each other (*t* test).

**Table 2:** Estimates of prevalence of illness from Illness Mapping and household survey

**Proportion of ailing persons (last month) obtained from the Illness Mapping**			**Proportion of ailing persons (last month) obtained from the household survey**
**Male and female groups combined**	**Male groups only**	**Female groups only**	
**(±SE**^**a**^**)**	**(±SE**^**a**^**)**	**(±SE**^**a**^**)**	
25.9% (±3.6%)	24.5% (±4.8%)	28.5% (±5.4%)	31.4%
	p = 0.587^b^		

^a^*SE* = Standard Error.

^b^ Test of significance between male and female groups (*t*-test).

### Types of illnesses

The proportion of acute and chronic illnesses in the Illness Mapping and the household survey data is shown in Table 3. Acute illnesses represented most of the morbidity under both counts (76.9% of all illnesses based on the Illness Mapping compared to 69.2% derived from the household survey). Chronic illnesses were 20.1% and 16.6% respectively. The proportion of accidents in the Illness Mapping (2.0%) was lower than that reported in the household survey (5.0%). There were fewer undefined illnesses in the Illness Mapping than in the household survey (1% vs. 9.1%).

**Table 3:** Estimates of types of illness from Illness Mapping and household survey

	**Illness types as share of illnesses:**			
	**Acute**	**Chronic**	**Accidents**	**Undefined**
Data obtained from the Illness Mapping	76.9%	20.1%	2.0%	1.0%
Data obtained from the household survey	69.2%	16.6%	5.0%	9.1%

Note: The above percentages for illness types were calculated for all groups together. Standard errors for these values are therefore not available.

### Hospitalizations

The Illness Mapping estimate of incidence of hospitalization was 1.1% (±0.4) and the household survey estimate was 2.6% (Table 4). Data from the household survey gave a much higher estimate than the Illness Mapping. The difference was significant and material even after taking the standard errors into account.

**Table 4:** Estimates of incidence of hospitalization from Illness Mapping and household survey

**Percentage of hospitalized persons (last year) obtained from the Illness Mapping**			**Percentage of hospitalized persons (last year) obtained from the household survey**
**Male and female groups combined**	**Male groups only**	**Female groups only**	
**(±SE**^**a**^**)**	**(±SE**^**a**^**)**	**(±SE**^**a**^**)**	
1.1% (±0.4%)	1.6% (±0.8%)	0.5% (±0.1%)	2.6%
	p = 0.213^b^		

^a^*SE* = Standard Error.

^b^ Test of significance between male and female groups (*t*-test).

### Deliveries

Data on incidence of deliveries and on percentage of hospital deliveries is presented in Tables 5 and 6. We found very good agreement between the Illness Mapping data and the household survey data on incidence of deliveries: 3.9% (±0.4) in the Illness Mapping data for all groups combined and 3.9% in the HH survey.

**Table 5:** Estimates of incidence of deliveries from Illness Mapping and household survey

**Number of deliveries per 100 persons (last year) obtained from the Illness Mapping**			**Number of deliveries per 100 persons (last year) obtained from the household survey**^**b**^
**Male and female groups combined**	**Male groups only**	**Female groups only**	
**(±SE**^**a**^**)**	**(±SE**^**a**^**)**	**(±SE**^**a**^**)**	
3.9% (±0.4%)	4.4% (±0.7%)	3.4% (±0.6%)	3.9%
	p = 0.293^c^		

^a^*SE* = Standard Error.

^b^ Based on the reported number of children less than or equal to 1 year in the household.

^c^ test of significance between male and female groups (*t*-test).

**Table 6:** Estimates of percentage of hospital deliveries from Illness Mapping and household survey

**Percentage of hospital deliveries obtained from the Illness Mapping**			**Percentage of hospital deliveries obtained from the household survey**
**Male and female groups combined**	**Male groups only**	**Female groups only**	
**(±SE**^**a**^**)**	**(±SE**^**a**^**)**	**(±SE**^**a**^**)**	
61.0% (±5.4%)	67.3% (±7.8%)	55.4% (±7.3%)	51.4%
	P=0.275^b^		

^a^*SE* = Standard Error.

^b^ Test of significance between male and female groups (*t*-test).

The Illness Mapping estimate of hospital or institutional deliveries was 61.0% (±5.4) for all groups combined, while the household survey estimate was 51.4% (Table 6). The two data series were within the good fit limit, but results reported by the female groups were in closer agreement (very good fit).

### Cost and time comparison between household survey and Illness Mapping

Table 7 gives a record of the time and human resources required for the household survey of 1,000 households compared to the Illness Mapping for 35 groups. The comparison is limited to the core activities related to the two methods, since the exact related costs could presumably be context dependent (salaries, traveling conditions, accommodations, will be different in different locations). The table shows that Illness Mapping represented a reduction of 59% in work-days, i.e. requires less time and less costs than conducting a household survey.

**Table 7:** Number of working days required for Illness Mapping and household survey

	**Illness Mapping**	**Household survey**
Preparation (including translation of tools, training of interviewers and pre-test)	3 days	8 days
Field work (with 1 supervisor and 4 or 5 interviewers)	18 days	30 days
Data entry (1 person)	1 day	20 days
Data cleaning and analysis (1 person)	8 days	14 days

## Discussion

In this study we set out to develop a reliable method that may in future enable us to access the necessary data for the establishment of a micro health insurance in low income rural communities where data would not be available otherwise. The objective before us was to find a way to overcome the two constraints associated with data sourcing through household survey, namely, the cost and time required. The Illness Mapping method we describe here seems to meet this objective. The information given in Table 7 illustrates the advantage of the Illness Mapping method in terms of human resources and time required, which obviously translate into differences in costs (e.g. salaries, travel, accommodation etc.).

The design of an insurance product requires estimates of the prevalence/incidence of the events covered by the insurance. Our previous studies showed that: (i) the incidence of illness episodes, and prevalence of hospitalizations and delivery is strongly context-dependent and varies across locations even in the same country [19] making it necessary to obtain local data. (ii) Prospective clients of health insurance in rural India are exposed to hardship financing not only in cases of hospitalizations but also in cases of outpatient treatment and in deliveries [4]. In fact, this is even more pronounced in case of chronic illnesses [6]. (iii) When expressing their priorities regarding benefits that should be covered by insurance, prospective clients expressed a clear wish to include both inpatient and outpatient benefits [34,37]. It is thus clear that the information obtained through Illness Mapping regarding the prevalence/incidence of prioritized cost generating events is essential for the design and pricing of context-relevant health insurance.

We followed a strategy of soliciting local information from groups rather than from individuals. We were inspired by group techniques, assuming that the small cosmos of a village community could be captured through harvesting the knowledge that is readily available to its inhabitants free of charge. Having failed to find a ready-made suitable method in the published literature, we opted to utilize a combination of established methods and adapt them to our settings. Group approaches such as the Delphi and NGT have been used successfully and with high accuracy for business forecasting as well as for public policy [38,39]. We adopted the criteria for resourcing quantitative information from qualitative non-interacting groups such as Delphi and NGT [22,26], and modified those to take account of the advantages of interactive group situations in which the discussions are moderated and facilitated rather than left to chance (as often happens in exploratory brainstorming groups or focus groups [28]). Such structured group methods are based on the principle of collective intelligence [40], or group intelligence that emerges through managed consensus decision making [31].

Our method was based on small group discussions with people who were marginally literate and numerate, but nonetheless experts or valid representatives of their village communities. They were chosen (with the help of our partner NGO staff who had prior access to the village) for their social attributes and their knowledge of households in their own neighbourhood in the village. In each village we carefully identified such participants and facilitated their interaction to obtain estimates for the prevalence of illness for the entire village. Other key contacts in the village such as teachers, village head, and health workers could also be recruited to provide similar information if there were no prior links with the village.

We organized gender homogenous groups in each village to ensure that both men and women would be able to express themselves freely. We thought that women, who are usually caregivers, might be more familiar with illnesses than men. However we found no statistical difference between the estimates given by men’s and women’s groups. We found it more difficult to assemble men’s groups as men were usually away during the day. From this experience we infer that Illness Mapping could be extracted from interactions with either gender of respondents, and that women’s groups are likely to be easier to assemble than men.

Our method had to be adjusted to the field reality of low literacy which meant that written consensus and voting was not the best option and so we employed a strategy which involved everyone in a sequential and structured interaction. Our structure emerged from the motivation, explanations, and facilitation techniques that we used to encourage accurate recall and steer discussions towards final consensus.

We examined the potential of our new Illness Mapping method by comparing the results obtained with those derived through a household survey. We compared three parameters which are important for implementation of micro health insurance: (i) prevalence of illness for acute and chronic illnesses, both of which entail cost implications which can be much higher in the case of chronic illnesses [18], (ii) incidence of hospitalization, as this cost is included in most health insurance programmes, and (iii) incidence of deliveries, especially hospital deliveries. We found very good agreement between the two methods on incidence of deliveries, and good agreement on prevalence of illnesses (in the last one month) and on prevalence of acute and chronic illnesses, as well as on the share of deliveries in hospital.

We obtained a lower estimate of incidence of hospitalization from the Illness Mapping than from the household survey (1.1% (±0.4) from the first source versus 2.6% from the second source). This discrepancy could be the result of two types of memory effects that can lead to erroneous reporting by respondents: errors of omission and of telescoping [41]. While omission means forgetting or omitting to report an episode entirely, telescoping works in the opposite direction, i.e. the respondent remembers and reports an event as having occurred more recently than it actually had. The telescoping effect increases the total number of events reported in a given period. It has also been found that telescoping may be greater in face to face interviews as the presence of an interviewer and the face to face interaction may prod the respondent to give “too much rather than too little information” [41]. It is possible that the telescoping effect may have resulted in an *overestimation* of hospitalizations in our household survey. In contrast, hospitalizations may have been *underestimated* in the Illness Mapping method as the group members may have only been aware of the longer duration hospitalizations in their communities and those due to major procedures such as surgeries. They may have omitted the shorter and less severe hospitalizations. This view is supported by prior evidence that longer duration stays and surgeries are more positively associated with recall than other hospitalizations [42]. We do not have a definitive basis to determine which of these estimates is more pronounced, and only actual utilization data could indicate which estimate is the more accurate prediction.

Data obtained either from Illness Mapping or from a household survey would usually be treated by insurers with some reserve, as both methods are less reliable than actual claims data over a long period of time. The Illness Mapping did not, a-priori, show any difference on this count relative to the data obtained from the household survey. In insurance business, it is therefore common practice to include a safety loading in premium calculations, to account for errors in assumptions or inaccuracy of estimates.

The main advantage of the Illness Mapping method is that it is cheaper and faster to operate, and could replace a household survey for estimating morbidity and healthcare utilization, especially where local data is needed but not readily available. While we have tested this method in rural settings in India, we have no reason to think that it could not be equally effective in urban settings (e.g. slums), or in other countries. The estimates about morbidity and healthcare utilization are of course essential not only for insurance purposes, but also for health policy choices more generally. Limitations of this method include the need to establish good contacts with the study communities in order to identify the most suitable community experts. Secondly, high quality group facilitation is essential, by facilitators that must speak the local language and understand the local social settings (and probably be local). Finally, as the estimates obtained by both methods are predictive, one powerful way to evaluate the robustness of the estimates obtained would be to examine both Illness Mapping data and household survey data against actual claims data. Such a follow-up examination is needed to validate the accuracy of the Illness Mapping as a generally applicable alternative to household surveys for the data in question.

## Conclusions

The effort to introduce health insurance among low income persons in areas in the informal economy requires that the benefit packages as well as the premiums payable will be customized to local conditions. Evidence has shown that those local conditions are context-specific and that one-size-fits-all simply will not do. This customization therefore is contingent on obtaining at least some local data on such pieces of information as prevalence of illness, hospitalizations, chronic and acute illnesses, and deliveries. We have explored the Illness Mapping method on the assumption that it can deliver a cheaper and faster resourcing of the essential local data, at acceptably accurate levels. We have shown in this study that the results obtained through the Illness Mapping method were comparable to those obtained through household survey. We have also shown that obtaining these results costs less time and money than conducting a household survey. We therefore conclude that for as long as health insurance solutions must be adapted to context relevant conditions and that these differ from one location to the next significantly, the Illness Mapping method tested in this study and explained in this article may serve the purpose.

## Endnotes

^i^The following conditions were usually included: (i) acute: fevers, diarrheas, body pains, respiratory conditions (not including asthma/COPD), TB and skin problems; (ii) chronic: asthma/COPD, diabetes, hypertension, kidney diseases, and cardiovascular problems.

## Abbreviations

ASHA: Accredited social health activist; HHS: Household survey; IM: Illness mapping; NGO: Non-governmental organization; NGT: Nominal group technique; NSS: National sample survey; SE: Standard error.

## Competing interests

The authors declare that they have no competing interests.

## Authors’ contributions

DMD and RK were responsible for the study concept and design. MG was responsible for the Illness Mapping fieldwork and data management; EB was responsible for the household survey fieldwork and data management. MG, EB, RK and DMD analysed and interpreted the data. EB, MG and RK prepared the draft of the manuscript. All authors read, revised and approved the final version of the manuscript.

## Authors’ information

MG was a Post-Doctoral Fellow, and EB a Ph.D. student with the Institute of Health Policy and Management, Erasmus University, Rotterdam, while developing this article. This work represents part of the requirements for the PhD thesis of EB at Erasmus University.

DMD, in addition to acting as principal investigator on this grant within his position as hon. Professor at Erasmus University Rotterdam, also acted as supervisor of MG in the Post-Doctoral fellowship and EB in the PhD studies. He is also the Chairman of the Micro Insurance Academy.

## Pre-publication history

The pre-publication history for this paper can be accessed here:

http://www.biomedcentral.com/1471-2288/12/153/prepub

## References

[B1] World Development Indicators Retrieved 09/13, 2012 from [http://data.worldbank.org/data-catalog/world-development-indicators]

[B2] Karan AK Selvaraj S Why publicly-financed health insurance schemes are ineffective in providing financial risk protection Econ Polit Wkly 2012 47 11 61

[B3] Ma S Sood N A comparison of the health systems in China and India 2008 Santa Monica, USA: Rand Corporation, Center for Asia Pacific Policy

[B4] Binnendijk E Koren R Dror DM Hardship financing of healthcare among rural poor in Orissa India. BMC health services research 2012 12 1 23 10.1186/1472-6963-12-23 PMC3317855 22284934

[B5] Peters DH Yazbeck AS Sharma RP Ramana GNV Pritchett LH Wagstaff A Better health systems for India’s poor: Findings, analysis and options 2002 Washington (DC): World Bank

[B6] Binnendijk E Koren R Dror DM Can the rural poor in India afford to treat non-communicable diseases Tropical Medicine & International Health 2012 [Epub ahead of print] 10.1111/j.1365-3156.2012.03070.x 22947207

[B7] Lopez AD Mathers CD Ezzati M Jamison DT Murray CJ Global and regional burden of disease and risk factors, 2001: Systematic analysis of population health data Lancet 2006 367 9524 1747 1757 10.1016/S0140-6736(06)68770-9 16731270

[B8] World Health Organization Global status report on noncommunicable diseases 2011 2010 Geneva, Switzerland: World Health Organization

[B9] Ahuja R Health insurance for the poor in India 2004 New Delhi, India: Working paper No. 123, Indian Council for research on International Economic Relation (ICRIER)

[B10] Ahuja R Health insurance for the poor in India: An analytical study 2005 New Delhi, India: Working Paper No. 161, Indian Council for Research on International Economic Relations (ICRIER)

[B11] Bhat R Jain N Factoring affecting the demand for health insurance in a micro insurance scheme 2006 Ahmedabad: IIMA Working Papers WP2006-07-02, Indian Institute of Management

[B12] National Commission on Macroeconomics and Health Financing and delivery of health care services in India 2005 New Delhi: National Commission on Macroeconomics and Health Background Papers, Ministry of Health and Family Welfare, Government of India

[B13] Dror DM Radermacher R Khadilkar SB Schout P Hay FX Singh A Koren R Microinsurance: Innovations in low-cost health insurance Health Aff (Millwood) 2009 28 6 1788 1798 10.1377/hlthaff.28.6.1788 19887420

[B14] Doyle C Panda P Van de Poel E Radermacher R Dror DM Reconciling research and implementation in micro health insurance experiments in India: Study protocol for a randomized controlled trial Trials 2011 12 1 224 10.1186/1745-6215-12-224 21988774 PMC3248368

[B15] Dong H Mugisha F Gbangou A Kouyate B Sauerborn R The feasibility of community-based health insurance in Burkina Faso Health Policy 2004 69 1 45 53 10.1016/j.healthpol.2003.12.001 15484606

[B16] AC Nielsen ORG-MARG Pvt Ltd Needs and demands for healthcare and health insurance amng landless agriculture laborers in Burdwan district, West Bengal 2001 Kolkota, India: Final Report submitted to GTZ Technical Assistance Team, Health Sector Support, Kolkata, AC Nielsen ORG-MARG Pvt Ltd

[B17] Dror DM Why “one-size-fits-all” health insurance products are unsuitable for low-income persons in the informal economy in India Asian Economic Review 2007 49 1 47 56

[B18] Dror DM van Putten-Rademaker O Koren R Cost of illness: Evidence from a study in five resource-poor locations in India Indian J Med Res 2008 127 4 347 361 18577789

[B19] Dror DM van Putten-Rademaker O Koren R Incidence of illness among resource-poor households: Evidence from five locations in India Indian J Med Res 2009 130 2 146 154 19797811

[B20] National Sample Survey Organization: Morbidity, health care and the condition of the aged. 2006, Report No. 507 (60th round January-June National Sample Survey Organization, Ministry of Statistics and Programme Implementation 2004 New Delhi: Government of India

[B21] National Sample Survey Organization: Morbidity and treatment of ailments. 1998, Report No. 441 (52th round July 1995-June National Sample Survey Organization, Ministry of Statistics and Programme Implementation 1996 New Delhi: Government of India

[B22] Jones J Hunter D Consensus methods for medical and health services research BMJ 1995 311 7001 376 380 10.1136/bmj.311.7001.376 7640549 PMC2550437

[B23] Aurey JP Fonteneau R Dror DM, Preker AS Local consensus and estimates of medical risk Social Reinsurance, A new approach to sustainable community health financing 2002 Geneva: The World Bank and the International Labour Organisation 187 222

[B24] Woudenberg F An evaluation of Delphi Technol Forecast Soc Chang 1991 40 2 131 150 10.1016/0040-1625(91)90002-W

[B25] Sample JA Nominal Group Technique Sample JA, Nominal group technique: An alternative to brainstorming J Ext 1984 22 2

[B26] Van de Ven AH Delbecq AL The effectiveness of nominal, Delphi, and interacting group decision making processes Acad Manag J 1974 17 4 605 621 10.2307/255641

[B27] Van de Ven AH Delbecq AL Nominal versus interacting group processes for committee decision-making effectiveness Acad Manag J 1971 14 2 203 212 10.2307/255307

[B28] Kitzinger J Qualitative research: Introducing focus groups BMJ 1995 311 7000 299 302 10.1136/bmj.311.7000.299 7633241 PMC2550365

[B29] Bouchard TJ Personality, problem-solving procedure, and performance in small groups J Appl Psychol 1969 53 1 (supplement 1 1 295797825

[B30] Bouchard TJ Training, motivation, and personality as determinants of the effectiveness of brainstorming groups and individuals J Appl Psychol 1972 56 4 324 331 5046062 10.1037/h0033028

[B31] Hart S Boroush M Enk G Hornick W Managing complexity through consensus mapping: Technology for the structuring of group decisions Acad Manag Rev 1985 10 3 587 600

[B32] Lowry PB Research on process structure for distributed, asynchronous collaborative writing groups 2002 Dallas: Proceedings of the 8th Annual Americas Conference on Information Systems 2172 2179

[B33] Som RK Practical sampling techniques 1996 2 New York: Marcel Dekker

[B34] Danis M Binnendijk E Vellakkal S Ost A Koren R Dror DM Eliciting health insurance benefit choices of low income groups Econ Polit Wkly 2007 42 32 3331 3339

[B35] National Sample Survey Organization Household Consumer Expenditure in India, 2006–07 2008 New Delhi: Report No. 527, National Sample Survey Organization, Ministry of Statistics and Programme Implementation, Government of India

[B36] National Sample Survey Organization Level and pattern of consumer expenditure, 2009–2010 2011 New Delhi: Report No. 538, National Sample Survey Organization, Ministry of Statistics and Programme Implementation, Government of India

[B37] Dror DM Koren R Ost A Binnendijk E Vellakkal S Danis M Health insurance benefit packages prioritized by low-income clients in India: three criteria to estimate effectiveness of choice Soc Sci Med 2007 64 4 884 896 10.1016/j.socscimed.2006.10.032 17141931

[B38] Basu S Schroeder RG Incorporating judgments in sales forecasts: Application of the Delphi method at American Hoist & Derrick Interfaces 1977 7 3 18 27 10.1287/inte.7.3.18

[B39] Hilbert M Miles I Othmer J Foresight tools for participative policy-making in inter-governmental processes in developing countries: Lessons learned from the eLAC Policy Priorities Delphi Technol Forecast Soc Chang 2009 76 7 880 896 10.1016/j.techfore.2009.01.001

[B40] Surowiecki J The wisdom of crowds 2004 USA: Double Day (Random House Inc)

[B41] Sudman S Bradburn NM Effects of time and memory factors on response in surveys J Am Stat Assoc 1973 68 344 805 815 10.1080/01621459.1973.10481428

[B42] Harlow SD Linet MS Agreement between questionnaire data and medical records: The evidence for accuracy of recall Am J Epidemiol 1989 129 2 233 248 2643301 10.1093/oxfordjournals.aje.a115129

